# Contegra conduit for reconstruction of the right ventricular outflow tract: a review of published early and mid-time results

**DOI:** 10.1186/1749-8090-3-62

**Published:** 2008-11-18

**Authors:** Aristotle D Protopapas, Thanos Athanasiou

**Affiliations:** 1 Department of Biosurgery and Surgical Technology, Imperial College London, St. Mary's Hospital, London, W2 1 NY, UK

## Abstract

**Objective:**

The valved conduit Contegra (bovine jugular vein) has being implanted for more than 7 years in the right ventricular outflow tract and it is noted that the available reports have been mixed. The aim of this study is to review the reported evidence in the literature.

**Methods:**

Search of the relevant literature for the primary endpoints of operative mortality and morbidity and secondary endpoints of follow-up haemodynamic performance including severe stenosis, regurgitation and need for reintervention are presented.

**Results:**

We selected and analysed 17 series including 767 patients. Commonest indication was Fallot's tetralogy. Operative mortality was 2.6%. Operative morbidity was 13.9%. In follow-up, the incidence of intraconduit stenosis was 10.9% (incidence of stenosis for the 12 millimetre conduit was 83.3% in one series) and that of at least moderate regurgitation was 6.3%.

The aspirin users had a stenosis incidence of 10.5% compared to the non-users had a stenosis incidence of 9.6%.

**Conclusion:**

A dissent on the performance of the Contegra is discussed, while results are satisfactory in the majority of studies apart for the smallest conduits (12 and 14 millimetre), suggesting an association to compromised run-off. The role of aspirin as antithrombotic modulator remains controversial.

## Introduction

The refinement of congenital cardiac surgical procedures requiring reconstruction of the right ventricular outflow tract (RVOT) (or substitution for the autograft pulmonary valve in Ross procedure) has led to an increased need for valved conduits, especially for neonates and small infants.

The haemodynamic performance and long-term complications of xenograft, composite grafts and homografts have been far from ideal. In view of the rising demand for conduits (especially of small internal diameter) and the decrease in homograft availability, alternative options have been sought.

A new solution popularised in Europe and of late in the USA and Canada is a valved conduit xenograft, the Contegra (formerly Venpro, Medtronic Inc, Minneapolis, MN, USA). Derived from bovine jugular vein, it incorporates the native tri-leaflet valve and a natural sinus. The prosthesis is preserved in buffered low concentration glutaraldehyde in order to maintain the leaflet flexibility. Contegra conduits are availble with internal diameter of 12,14,16,18, 20 and 22 millimeters (mm). Theoretical advantages include structural continuity and 'off-the-shelf' availability. An inherent feature of the Contegra is the fact that being derived from the venous circulation and has the morphological characteristics of a 'vascular' valved conduit under conditions of low pressure similar to the pulmonary vascular tree (and not the higher pressure and hemodynamic condition of the systemic circulation). Therefore its use is not licenced for the left ventricular outflow tract.

A number of studies have reported on this conduit. It appears that some surgeons are concerned with its performance, while others are satisfied. In order to draw some further practical conclusions, we have systematically reviewed the literature in order to assess the existing cumulative peri-operative data and postoperative performance.

## Methods

Ethical issues were not raised, therefore ethical approval was not sought. No conflict of interest is declared.

We performed a literature search (PubMed) for studies reporting on Contegra, published between 1965 and 31 March 2007): Searching keyword was "Contegra" limited to "human subjects." Articles were also identified using the function "related articles" in PubMed and cross-validated by hand search. No language restrictions were applied.

We performed summary descriptive statistics on pooled data:Primary outcomes discussed were surgical mortality and morbidity and secondary outcomes as follow-up haemodynamic performance including: intra-conduit pressure difference recordings, severe conduit stenosis and regurgitation as also need for reintervention. Special care was given to include data on conduit specific reintervention incidence during the follow-up period. Postoperative aspirin strategy was also assessed. Denominators were related to actual data. Missing data were not defaulted. Where follow-up data were sought, we opted for a period prevalence denominator of patient-years and haemodynamic assessment was extracted focusing at the time point of measurement. Due to inherent heterogeneity we did not attempt statistical inference.

### Data and analysis

We identified and considered 22 publications [1-22]. Five of them included data from others [2] from [13,3] from [14] and [15,6] from [16,11] from [19] with apparently overlapping cohorts, so we utilized data from the largest ones [2,3,6,11].

Two papers from the same country [2,6] presented equal number of subjects with partially overlapping periods of observation. The units where surgery took place were not stated in either. The rest of their data being dissimilar, we concluded that the two series had to be entered to the database separately and that the same number of patients appears plainly a coincidence.

Thus we accumulated data from 17 series [1-12,17,18,21-23].

## Results

### 1. Demographics And Pathologies

Data from 767 patients were reported in total in these 17 studies (ranging from 6 to 108 per study). Each patient received a single Contegra with the exception of two case that had two each [1,20].

Mean age was 7.6 years (range 2 days-55 years). Mean weight was 23.1 Kg (range 2.8–125 Kg). The commonest indications for implantation of Contegra was Fallot's Tetralogy (198 patients), Arterial Trunk (Truncus, 91 patients), Ross procedure (80 patients), Pulmonary Atresia (50 patients), Double Outlet/Inlet Right Ventricle (40 patients), Transposition of Great Arteries (29 patients).

### 2. Operative Outcomes

We sought the standardised surgical outcomes according to published guidelines[23].

Mortality

There were 20 deaths reported in 766 operations. The cumulative Mortality was thus 2.6%.

Morbidity

Morbidity is presented in Table 1. Morbidity data were individually and clearly reported in 8 series with 330 patients [[3-5,8,10-12] and [23]]: 46 patients suffered one or more of 54 complications. Cumulative operative morbidity was thus calculated to 13.9%.

**Table 1:** Contegra™ 1999–2007- perioperative complications

Type	Number of incidents
Bleeding	5

Neurological	3

Sepsis	4

Respiratory	13

Thrombosis	2

Renal	2

Cardiac	13

Other	12

Total	54

### 3. Follow-Up

584 patients had being followed up for 22 months in average (range 1–56) rending the total calculated follow-up to 573 patient-years.

Echocardiography was utilised for routine interval follow-up.

Severe Stenosis and timepoint of follow-up is presented in Table 2.

**Table 2:** Contegra™ 1999–2007, follow-up pressure difference and anticoagulation strategy, re-interventions in detail

reference	N	peak ΔP in mmHg	mean ΔP in mmHg	Anticoagulation strategy	Percutaneous	Surgical	Total Reinterventions
[1]	55	unknown	unknown	nil	17	5	22

[2]	67	17	8	aspirin for 6 months	2	1	3

[3]	100	18	unknown	nil	0	4	4

[4]	29	unknown	unknown	Aspirin until weighing 5–6 kg	2	1	3

[5]	20	unknown	13 ± 4.8	Heparinisation and aspirin	0	0	0

[6]	67	unknown	11	nil	0	1	1

[7]	30	unknown		nil	0	2	2

[8]	15		11.1 ± 4.5	nil	0	0	0

[9]	6	8.5	24.5 ± 13.1	aspirin for 3 months max	0	5	5

[10]	40	'increased' in 2- unknown otherwise		nil	0	3	3

[11]	38	unknown		nil	0	0	0

[12]	12	unknown	12.9 ± 12.3	nil	6	3	9

[17]	60	unknown		aspirin for 6 months	6	1	7

[21]	62	unknown	14	aspirin for 3 months	0	0	0

[18]	76	unknown		aspirin	4	0	4

[22]	78	23	unknown	Heparinisation and aspirin	19	2	21

					56	28	84

N: number of patients followed-up

ΔP: Pressure Difference, commonly reported as 'gradient'

Hg: Mercury

PA: pulmonary artery(-ies)

Average pressure differences (commonly yet erroneously [24] termed 'gradient') were: peak 17 mm Hg (millimetres of Mercury) (range 8.5–27) and mean 8.2 mmHg (range 7–13). Incidence of intraconduit stenosis, was 10.9% necessitating 56 interventional and 28 surgical procedures.

Routine postoperative aspirin administration was explicitly stated in six series [2,5,9,17,21,22]. The authors of another series [4] reported adding post discharge aspirin empirically in neonates (10 mg/day till the patient reaches 5–6 Kg of body weight) after two thrombotic events in their cohort. Routine aspirin administration is not mentioned in the other series.

The aspirin users had a stenosis incidence of 10.5%. The non-users had a stenosis incidence of 9.6%.

Regurgitation-Insufficiency(Table 3)

**Table 3:** Contegra™ 1999–2007: postoperative moderate or severe regurgitation/insufficiency in conduit

Reference Number	Degree Of Regurgitation/Insufficiency		
	
	III(moderate)	III-IV(moderate to severe)	IV (severe)
1	0	9	

2		0	

4	1	0	

5		0	

6		0	

8		0	

10		0	

11		0	

7		0	3

12		0	

17		13	3

21		0	

20		0	

21		12	

22		2	2

Incidence of at least moderate regurgitation was 6.3% as assessed by echocardiography in standardised methodology[25].

## Discussion

Quality control in surgery motivates to good operative outcomes [21] but also to long-term results, particularly in surgery for congenital heart disease. We have sometimes seen a 'euphoric' description of operative results but disappointing long-term outcome.

A dissent on the performance of the Contegra is noted: The titles of three of the largest series [1,2,20] were contradictory: direct unequivocal negative message in the first [that was debated strongly in discussion by the audience [1] and enthusiasm in the other two [2,20]. Meyns [1] had apparently stopped utilizing Contegra! The difference in opinions may be partially explained by the heterogeneity of the series, as expected from an intervention applicable to premature neonates as well as adults for a constellation of indications.

The experience of Meyns is isolated and rather baffling! If it is excluded, the results are encouraging overall but the follow-up is short. The reason for the high incidence of distal stenosis in Meyns' group remains obscure although the non-use of aspirin may contribute.

The few data on pressure differences are exemplary for the limited data available for all prostheses in vivo. We have to insist that the ubiquitous term 'pressure gradient' is inaccurate when measured in units of pressure and was perceived as pressure difference [24].

The definition of graft stenosis is extremely varying (especially if taken into account the large number of young children where low gradient has a completely different significance than in an adult): higher stenosis rate has been shown in smaller- size patients and conduits.

Echocardiography for estimation of pressure difference is the most common technique since it is easy to apply and relatively of low cost. However, having a large number of complex defects, the question of specificity and sensitivity is important.

Having a relative low number of patient-years of follow-up could reflect a high mid-term mortality or incomplete follow-up data dies.

A contentious issue is that of post discharge aspirin [[1], see pages 838–40]. (Table 2). Out of the present data, a conclusive answer to whether aspirin prevents graft thrombosis and stenosis is rather difficult. A definitive conclusion would require a randomised prospective trial in order to avoid confounding biases. The feasibility and the ethics of such a study are however also debatable, in the light of the experience of Tiete [4]. It appears reasonable to 'aspirinise' the recipients of Contegra in the absence of contraindications given the known prophylactic value of aspirin in small conduits as coronary bypass.

The choice of the internal diameter of conduit is related to the anatomy and size of the patient (table 4). Most surgeons agree that the largest possible diameter conduit should be utilised. It is impressive that the incidence of stenosis in the smallest 12 mm conduit was 83.3% in one trial with diligent follow-up of two years[1]. Interrupted suture line could be considered as a potential solution to the distal anastomotic stenosis but is obviously upon to surgeon's preference to apply this technique due to increased risk of bleeding.

**Table 4:** Contegra™ 1999–2007: median size of conduit and follow-up

median size mm	stenosis at follow-up	reintervention or reoperation for conduit failure at follow-up	regurgitation at follow-up
20	49% ± 8(28 months)	41% (22.7 ± 10 months)	16%(22.7 ± 10 months)

20	unknown	10.4%(26.4 months)	0%(26.4 months)

14		10 (1.1 ± 1.1 years)	

14		11.6%(1 year)	3.4%(10.2 ± 6.4 months)

22	0(13.8 ± 9.1 months)	0(13.8 ± 9.1 months)	0(13.8 ± 9.1 months)

unknown	3%(10 ± 2.8 months)	3%(10 ± 2.8 months)	

14	12.5%(3 months)	12.5%(4.3 ± 2 months)	12.5%(3 months)

unknown		6.3%(18.5 ± 6.9 months)	0%(18.5 ± 6.9 months)

unknown		0%(4 ± 2.7 months)	

16	unknown	unknown	unknown

16	15.8%(18 ± 5 months)	13.1%(18 ± 5 months)	unknown

16	0(8.08 months)	unknown	8.3%(8.08 months)

14	11.7%(14 months)	34%(38 months)	26.7%(14 months)

unknown			

16	18%(3 years)	6.9%(3 years)	36%(3 years)

unknown		29.5%(4 years)	16.5%(4 years)

unknown	0%(28.1 ± 17.1 months)	0%(28.1 ± 17.1 months)	0%(28.1 ± 17.1 months)

Complications were also commoner in double outlet right ventricle and pulmonary atresia, possible because of variable run-off in distal pulmonary vasculature. The excellent results reported on a series of 20 Ross procedures [5], where the run-off at the normal pulmonary vessels is assumed universally good.

## Conclusion

A dissent on the performance of the Contegra is noted: This lack of consensus regarding the best conduit for RVOT reconstruction reflects the fact that no "perfect" solution is available.

It is important to formulate an evidence- base opinion on the available technologies and we hope that our review will address this.

## Limitations of the study

Although every effort was made to extract data that can be pooled and tabulated, the heterogeneity of demographics, casemix and reporting studies performed in different institutions, with different methodologies, populations and time frames was considered an obstacle in inferential statistics; therefore we report descriptive statistics. Comparison between the groups was difficult, especially as data on conduit sizes were missing from the largest series in particular.

We understand that the heterogeneity data may be interpreted in a number of ways equally valid to the one the authors chose here.

## Competing interests

The authors declare that they have no competing interests. AP has inserted the Contegra and managed patients with previous Contegra insertion.

## Authors' contributions

AP drafted the first manuscript; TA corrected and co-authored the final manuscript.
